# The Impact of International Classification of Disease–Triggered Prescription Support on Telemedicine: Observational Analysis of Efficiency and Guideline Adherence

**DOI:** 10.2196/56681

**Published:** 2024-10-25

**Authors:** Tarso Augusto Duenhas Accorsi, Anderson Aires Eduardo, Carlos Guilherme Baptista, Flavio Tocci Moreira, Renata Albaladejo Morbeck, Karen Francine Köhler, Karine de Amicis Lima, Carlos Henrique Sartorato Pedrotti

**Affiliations:** 1 Telemedicine Department Hospital Israelita Albert Einstein São Paulo Brazil; 2 Digital Platform Hospital Israelita Albert Einstein São Paulo Brazil

**Keywords:** telemedicine, clinical decision support systems, electronic prescriptions, guideline adherence, consultation efficiency, International Classification of Disease–coded prescriptions, teleheath, eHealth

## Abstract

**Background:**

Integrating decision support systems into telemedicine may optimize consultation efficiency and adherence to clinical guidelines; however, the extent of such effects remains underexplored.

**Objective:**

This study aims to evaluate the use of *ICD* (*International Classification of Disease*)-coded prescription decision support systems (PDSSs) and the effects of these systems on consultation duration and guideline adherence during telemedicine encounters.

**Methods:**

In this retrospective, single-center, observational study conducted from October 2021 to March 2022, adult patients who sought urgent digital care via direct-to-consumer video consultations were included. Physicians had access to current guidelines and could use an *ICD*-triggered PDSS (which was introduced in January 2022 after a preliminary test in the preceding month) for 26 guideline-based conditions. This study analyzed the impact of implementing automated prescription systems and compared these systems to manual prescription processes in terms of consultation duration and guideline adherence.

**Results:**

This study included 10,485 telemedicine encounters involving 9644 patients, with 12,346 prescriptions issued by 290 physicians. Automated prescriptions were used in 5022 (40.67%) of the consultations following system integration. Before introducing decision support, 4497 (36.42%) prescriptions were issued, which increased to 7849 (63.57%) postimplementation. The physician’s average consultation time decreased significantly to 9.5 (SD 5.5) minutes from 11.2 (SD 5.9) minutes after PDSS implementation (*P*<.001). Of the 12,346 prescriptions, 8683 (70.34%) were aligned with disease-specific international guidelines tailored for telemedicine encounters. Primary medication adherence in accordance with existing guidelines was significantly greater in the decision support group than in the manual group (n=4697, 93.53% vs n=1389, 49.14%; *P*<.001).

**Conclusions:**

Most of the physicians adopted the PDSS, and the results demonstrated the use of the *ICD*-code system in reducing consultation times and increasing guideline adherence. These systems appear to be valuable for enhancing the efficiency and quality of telemedicine consultations by supporting evidence-based clinical decision-making.

## Introduction

Telemedicine increasingly serves as a primary point of entry into the health care system for patients, particularly in urgent care scenarios [1]. Physicians providing digital consultations are tasked with maximizing efficiency in terms of encounter duration while ensuring that prescriptions issued adhere to established guidelines, which is a crucial component of the cost-effectiveness and quality of telemedicine services [2].

The role of prescription decision support systems (PDSSs) is critical in the digital health care environment [3]. Within electronic health records (EHRs), PDSSs can streamline the amount of time that clinicians spend navigating complex medical terminology [4]. Research indicates that standardizing data input can enhance routine documentation in medical records [5]. Apart from time efficiency, EHRs have been linked to improved care quality and increased compliance with clinical guidelines [6]. Although EHR adoption is associated with significant challenges, the development of strategies to facilitate their use is gradually progressing [7]. To date, however, research exploring voluntary PDSS use and its impact on the outcomes of direct-to-consumer telemedicine consultations for acute conditions has been lacking.

We propose that physicians’ adoption of a PDSS will demonstrate both a reduction in the time needed to deliver care and increased adherence to clinical guidelines. This study explored the relationships between telemedicine use and *ICD* (*International Classification of Diseases*)-triggered PDSS scores, consultation duration, and guideline adherence.

## Methods

### Study Design and Participants

A single-center retrospective study is conducted at the Telemedicine Center of Hospital Israelita Albert Einstein in São Paulo, Brazil. The data were collected by physicians at the Telemedicine Center, which ensured secure data storage. All authors contributed to the initial draft of the study and conducted a thorough examination of the complete data set, to which they had full access. The paper was exclusively written by the named authors, without contributions from nonauthors. All data analyses were conducted internally by the supervisory team of the Telemedicine Center. All authors collectively decided to submit this paper for publication and also supported the authenticity and integrity of the reported data.

The study included patients aged 16 years and older who voluntarily accessed digital direct-to-consumer care from October 2021 to March 2022. We considered all patients who were presented with any medical condition for inclusion in this study. The only exclusion criterion was the occurrence of connection issues that precluded the creation of medical records; these patients were excluded because they did not undergo a complete medical evaluation and were consequently not documented in the institution’s database.

### Ethical Considerations

The protocol for the study, which is known as the “Tele AUTOMATION” trial, and a consent waiver (based on an analysis of anonymized retrospective data from routine care) were approved by the Hospital Israelita Albert Einstein Review Board (registration: CAAE 69981423.6.0000.0071). Institutional digital archives, which were not linked to any external financial support, served as repositories for all of the data related to this study. The collected data were treated confidentially and protected by strict security measures, in accordance with the internal data protection policies of the Hospital Israelita Albert Einstein. All stages of the study involving privacy and personal data protection were conducted in accordance with Brazil’s General Data Protection Law (LGPD). No compensation was provided to participants.

### Telemedicine Consultations

Telemedicine consultations were conducted over the internet using proprietary videoconferencing software and EHRs. All participating physicians were board-certified and had additional training in both telemedicine and emergency medicine. The Telemedicine Center provided streamlined access to up-to-date clinical guidelines. Medical information was recorded in EHRs, which featured a specific field for the primary *ICD-10* (*International Statistical Classification of Diseases, Tenth Revision*) diagnosis.

### Decision Support and Guideline-Directed Prescribing

A suite of 26 international guidelines adapted for telemedicine was available for immediate reference during the consultations. These guidelines were structured to suggest appropriate prescriptions, as outlined in Multimedia Appendix 1. Physicians received training on how to effectively consult these guidelines, with the aim of aligning prescriptions with scientific evidence, resource efficiency, side effect mitigation, and overall service safety. In 2021, a voluntary feature allowing prescription autopopulation was introduced, providing shortcuts to medications listed in guidelines corresponding to the *ICD-10*, which is central to patient care. With a single keystroke, the autopopulation feature filled prescription fields with medications, dosages, administration routes, and durations as indicated by the relevant guidelines. This new functionality was introduced during the testing period, and no further adaptations were made to the system. By January 2022, this functionality was fully integrated into the medical record system, and physicians were instructed on its use according to clinical findings. Prescription adaptations for comorbidities were tailored based on clinical judgment and evaluated on an individual basis.

Patient and physician acceptance was not directly assessed. The functionality was made available for voluntary use. Although system adoption was not directly measured, a substantial portion of prescriptions were issued using the decision support system, allowing us to infer that a significant number of physicians accepted this strategy.

### Data Extraction and Adherence to Institutional Protocols

The data from each TM encounter were extracted from the institution’s medical record database. The *ICD-10* code associated with each telemedicine consultation was used to cross-reference the institutional protocol. Prescriptions issued by the telemedicine physicians were compared to the protocol’s medication list using data from the medication prescription field in the telemedicine consultation record.

First, the *ICD-10* code from each telemedicine consultation was used to identify the proper institutional protocol. Then, the set of medications provided in the protocol was compared with the medications prescribed by the telemedicine physicians as documented in the dedicated fields for prescriptions on the telemedicine consultation record. We added a binary feature (1=matching and 0=not matching) to the data set to denote matches between prescribed medication and institutional directives. After applying this procedure to all records in our data set, we were able to compute summary statistics to compare protocol adherence before and after autocomplete system implementation. Table 1 provides the main summary statistics for our data set.

**Table 1:** Summary statistics for the data set before and after implementation of the clinical decision support autocomplete system.

Data set feature (year)	2021	2022	Total
Consultations, n (%)	3873 (36.94)	6612 (63.06)	10,485
Patients, n (%)	3628 (37.62)	6174 (68.38)	9644
Physicians, n (%)	211 (72.76)	231 (79.65)	290
Medication prescriptions, n (%)	4497 (36.42)	7849 (63.58)	12,346
Mean encounter duration (in minutes), mean (SD)	11.23 (5.92)	9.49 (5.57)	10.13 (5.77)
*ICD-10*^a^ codes documented, n (%)	286 (65.15)	312 (71.07)	439

^a^*ICD-10*: *International Statistical Classification of Disease, Tenth Revision*.

### Statistical Analysis

The study analyzed a convenience sample of all patients who were consecutively registered during the defined period. Continuous variables are reported as the mean (SD) or as the median (IQR) ranges for descriptive purposes, whereas categorical variables are summarized as counts and percentages. To test for normality in the distribution of our sample, we used the Kolmogorov-Smirnov test. The Mann-Whitney *U* test was used for continuous variables that were not normally distributed. A *P* value less than .001 indicated statistical significance and 95 % CIs were calculated. All statistical analyses were conducted using IBM SPSS Statistics (version 22.0) for Windows.

## Results

Throughout the 6-month study period, we analyzed a total of 10,485 encounters with 9644 patients. Patient demographic data and the most common diagnoses are described in Table 2. These encounters resulted in 12,346 prescriptions being issued by 290 different attending physicians. Notably, some patients had multiple consultations within the study timeframe, and multiple prescriptions were occasionally dispensed during a single consultation; these prescriptions were not individualized. Before the implementation of the self-report prescription system, 4497 (36.42%) prescriptions were issued, which increased to 7849 (63.58%) after its implementation. A preliminary test of the system commenced in 2021; however, the system was not fully operational and made available for voluntary use by all physicians until January 2022. Following its implementation, the automated prescription feature was used in 5022 (40.67%) of the encounters.

During the brief trial period of the PDSS tool in 2021, a total of 261 (5.80%) prescriptions were made with electronic assistance, while 4236 (94.19%) prescriptions were issued without such assistance. Following the full deployment of the PDSS tool in 2022, the figures shifted significantly, with 5022 (63.98%) prescriptions being made with electronic assistance compared to 2827 (36.02%) without assistance.

**Figure 1:**
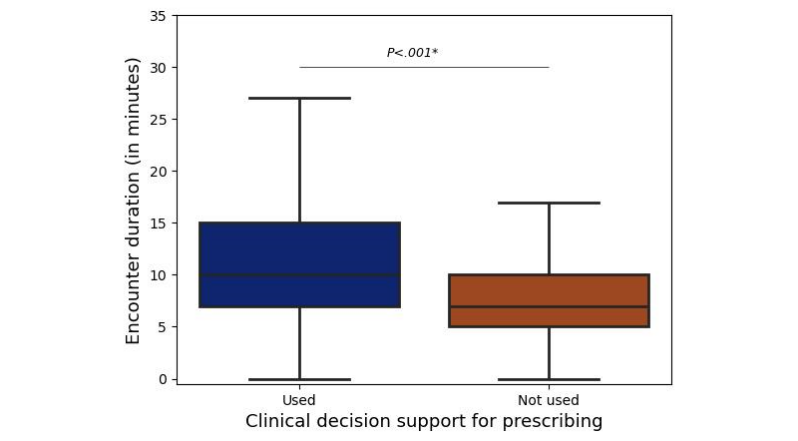
Comparison of consultation times with and without the use of autocomplete functionality for medical prescriptions revealed a statistically significant decrease in consultation time (*P<.001; 9.5, SD 5.5 minutes vs 11.2, SD 5.9 minutes; Mann‒Whitney U test=12,118,181.5).

**Figure 2:**
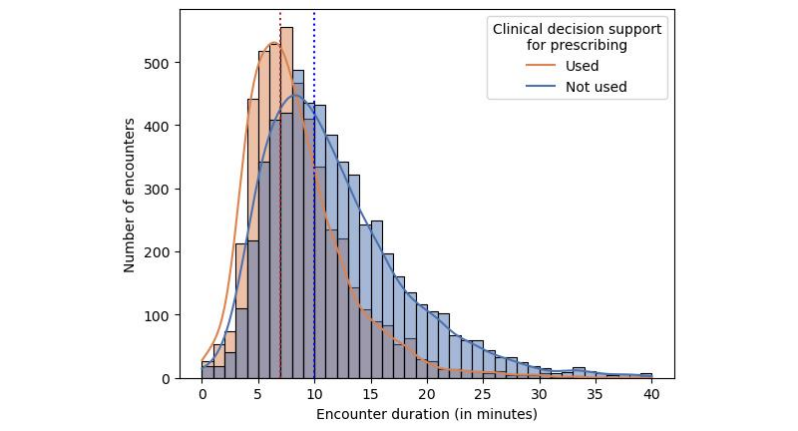
Distributions of consultation times and encounter frequency. The dotted vertical lines indicate the median values for each distribution (8 and 10 minutes, respectively).

The data demonstrated a significant reduction in consultation time after self-reporting: 9.5 (SD 5.5) minutes versus 11.2 (SD 5.9) minutes (*P*<.001; Mann-Whitney *U* test=12,118,181.5; Figure 1). Figure 2 shows that the decrease in the average consultation duration was correlated with service density, which was defined as the volume of services delivered by the center within a specified timeframe.

Regarding guideline adherence among the 12,346 medications prescribed, a substantial number of these medications (n=8683, 70.34%) were prescribed in accordance with guidelines. Notably, compared with manual prescription entry, the use of the automated filling system significantly increased guideline adherence (n=4697, 93.5% vs n=1389, 49.1%; *P*<.001; *z* score=45.24).

Regarding adherence to the institutional protocol for medical prescriptions, when the PDSS tool was not used, 1438 (50.87%) prescriptions deviated from the standard recommendations, while 1389 (49.13%) complied. Our analysis demonstrated that the application of the PDSS tool significantly improved adherence, with only 325 (6.47%) prescriptions failing to comply with the protocol, compared to 4697 (93.53%) that were aligned.

**Table 2:** Patient demographic data and the most common diagnoses.

Variable		2021-2022
Encounters		10,485
Patients		9644
**Sex, n (%)**		
	Male	6669 (69.15)
	Female	2875 (29.81)
	Not declared	100 (1.04)
Age (years), mean (SD)		0.0-92.0 (32.2)
**Most common diagnoses, n (%)**		
	J01, acute sinusitis	2627 (25.05)
	N30, cystitis	1839 (17.54)
	J03, acute tonsillitis	1271 (12.12)
	J02, acute pharyngitis	478 (4.56)
	J06.9, acute upper respiratory infection, unspecified	324 (3.09)

## Discussion

This study demonstrated that shortly after becoming available, most physicians opted to voluntarily use the decision support autocomplete functionality for medical prescriptions. Innovative approaches that facilitate the use of EHRs tend to be well received by medical practitioners [8]. The integration of new information technologies has transformed numerous aspects of health care and thus revolutionized care delivery within health systems [9], representing a significant advancement in digital health care, although certain challenges still need to be overcome. These challenges include poor interface designs, suboptimal performance, maintenance issues, overreliance, and dependency, all of which could jeopardize patient safety [10].

This study revealed an impressive rate of 94.7% adherence to recommendations, suggesting a potential improvement in the safety of telemedicine consultations with the use of autocomplete prescriptions. Guideline adherence is a measure of how well health care providers follow established clinical guidelines and protocols and is crucial for minimizing risks and ensuring optimal care quality and safety [11]. Notably, given that evidence suggests frequently deficient adherence with respect to in-person consultations, telemedicine shows promise as a method of care associated with better compliance [12-14].

The safety of prescribing practices and reductions in medication errors have been supported by evidence from electronic prescriptions selected through the use of standardized medication lists, codified instructions, and multimodal decision support [15]. Nonetheless, the prevalence of electronic prescribing errors can be high, with rates nearing 60%, particularly when considering incorrect field entries [16]. Previous studies have identified numerous potential error types in electronic prescriptions [17], and the electronic prescription strategy chosen has been noted to be a source of medication errors [18], which underscores the importance of continuing to improve electronic prescription systems.

The option for voluntary prescription autopopulation has several potential benefits for health care delivery. This approach may alleviate bureaucratic burdens associated with current medical practices, thereby enhancing communication with patients [19]. In high-demand settings, an autocomplete option can optimize consultation times as part of a broader management strategy [20]. Additionally, access to checklists for possible medication recommendations may reduce bias [21].

Our analysis revealed a 15.18% decrease in physicians’ average consultation time, from 1123 to 949 minutes, when the autocomplete feature was used, although reliance entirely on manual prescription entry was noted in 31% of consultations. Intriguingly, even with voluntary use, most prescriptions issued using the autocomplete function strictly adhered to medication recommendations, indicating high adherence to these recommendations. Prior to our study, no research had specifically investigated telemedicine quality based on guideline-directed prescribing facilitated by an autocomplete function. Accordingly, the establishment of high-quality telemedicine centers that continuously update management guidelines and develop strategies to meet policy requirements and deliver excellent remote medical consultations is imperative to enhance EHR systems. In addition to prescribing practices alone, administrators need to ensure the usability and appropriate implementation of coding for the autocomplete method to prevent misuse and maintain safety.

No specific security assessment was conducted. However, protocol adherence is recognized as a primary indicator of safety in care delivery, including referrals for in-person care when warning signs are identified. This study revealed enhanced adherence to guideline recommendations through the use of the self-report system, indirectly suggesting that increased safety was observed within this group.

Among the limitations of this study, the observed outcomes from the use of the clinical decision support system may not be solely attributable to changes in care practices. Instead, these outcomes might also reflect changes in the *ICD* codes selected by physicians, possibly for the convenience of prescribing. Moreover, the clinical support decision system may have encouraged excessive prescribing despite many of these conditions being manageable with nonpharmacological interventions, especially for those with mild symptoms. Notably, care quality assessed based solely on whether *ICD* codes matched medication recommendations may not effectively represent the true quality of care and may reflect decreased care quality, as previously mentioned. Another possible limitation was that the group that used decision support may have included patients with simpler diagnoses.

In this study, a PDSS was voluntarily used by physicians for most encounters. Decision support incorporation for prescription selection within EHRs, particularly in scenarios where policies are clearly established, may contribute to reducing consultation times and promoting high rates of adherence to guideline-directed prescriptions. This finding highlights the potential role of these systems in improving both the efficiency and quality of health care delivery, especially within telemedicine environments where prompt and precise decision-making is essential.

## Appendix

Multimedia Appendix 1 Compilation of ICD-10 codes with corresponding guideline-directed prescriptions. ICD-10: International Statistical Classification of Disease, Tenth Revision.

## Abbreviations

EHR: electronic health record
ICD: International Classification of Diseases
ICD-10: International Statistical Classification of Diseases, Tenth Revision
PDSS: prescription decision support system
