# Supplementary material for: The Impact of International Classification of Disease–Triggered Prescription Support on Telemedicine: Observational Analysis of Efficiency and Guideline Adherence
Source: JMIR Med Inform. 2024 Oct 25;12:e56681. doi: 10.2196/56681 (PMC11549578; doi:10.2196/56681)
Supplement: Multimedia Appendix 1 [file medinform_v12i1e56681_app1.docx]

**Multimedia Appendix 1**

**Table S1.** Compilation of *ICD-10*^a^ codes with corresponding guideline-directed prescriptions.

| *ICD-10* | Final diagnosis | Suggested prescription |
| --- | --- | --- |
| K12 | Recurrent oral aphthae | - In the absence of warning signs, recommend treatment with topical corticosteroids (hydrocortisone acetate twice a day until improvement for a maximum of 7 days) and, if necessary, a topical anesthetic (lidocaine ointment twice a day until improvement for a maximum of 7 days). |
| R51, G43, G44, G44.2 | Headache | - Combined analgesia: (1) dipyrone 1 g—take 2 tablets initially and then 1 tablet every 6 hours if pain persists; (2) naproxen 500 or 550 mg—take 1 tablet initially and then every 12 hours if pain persists. - Antinausea: (1) metoclopramide 10 mg—take 1 dose initially and then every 8 hours if nausea persists; OR (2) ondansetron 8 mg—take 1 tablet sublingually every 8 hours if nausea occurs. - Muscle relaxant: (1) cyclobenzaprine 5 to 10 mg—take daily, before bed, for 5 to 7 days. - Antimigraine: (1) dihydroergotamine 1 mg + dipyrone 350 mg + caffeine 100 mg—take 1 to 2 tablets initially and then, 1 additional tablet if no improvement is noted within 30 minutes; do not use more than 3 tablets on the same day; OR (2) naratriptan 2.5 mg—take 1 tablet at symptom onset; do not take more than 1 tablet on the same day without medical consultation. |
| N30 | Cystitis | - Empirical antibiotic therapy: (1) fosfomycin 3 g—take 1 sachet diluted in water as a single dose, preferably on an empty stomach, after emptying your bladder at night and just before sleep; OR (2) norfloxacin 400 mg—take 1 tablet every 12 hours for 3-7 days; OR (3) nitrofurantoin 100 mg—take 1 capsule every 6 hours for 5-7 days. - Analgesia: (1) phenazopyridine 200 mg—take 1 tablet every 8 hours for pain with urination for up to 3 days (urine may appear orange or reddish). (2) scopolamine 10 mg + dipyrone 250 mg—take 2 tablets no later than 8 AM for lower abdominal pain for up to 3 days. |
| S20, S30, S40, S50, S60, S70, S80, S90, T14 | Contusions | - Ketoprofen 100 mg orally every 12 hours for 3-5 days naproxen 500 mg + esomeprazole 20 mg orally every 12 hours for 3-5 days. - Dipyrone 1 g orally every 6 hours for 3-5 days. - Cyclobenzaprine 5 to 10 mg orally before bed for 3-5 days. - For those suffering from allergies: paracetamol 750 mg every 6 hours for 3-5 days. |
| I10, I15, O10, O12, O13, O14, O16, R03 | Hypertension | - Routine medication is not recommended. |
| L20, L21, L23, L24, L25, L30, L30.9 | Dermatitis | - For intense cases: betamethasone valerate 0.1% (do not use on the face or in folds)—apply to affected areas twice daily for up to 7 days. - For moderate cases: mometasone furoate 0.1%—apply to affected areas once a day for up to 14 days. - For the eye area, folds, or intimate areas: desonide cream 0.05% OR hydrocortisone 1%—apply to affected areas twice a day for up to 14 days. - Hypoallergenic, fragrance-free moisturizing cream—apply to affected areas several times a day until the condition improves. Choosing creams offering intensive hydration that are rich in lipids (examples: Bepantol Derma, Cetaphil Advanced, Fisiogel AI). |
| A09, K58, K59 | Diarrhea | - Combined analgesia: (1) scopolamine 10 mg + dipyrone 250 mg—take one tablet every 8 hours for cramps for up to 3 days. - Antinausea: (1) ondansetron 8 mg—take 1 tablet sublingually every 8 hours for nausea or vomiting for up to 3 days. - Antidiarrheal: a. Racecadotril 100 mg every 8 hours until diarrhea improves; OR loperamide 2 mg – Take two tablets initially and then one tablet after each episode of diarrhea; do not exceed eight tablets per day. - *Saccharomyces boulardii* 200 mg (capsules) – Take one capsule every 12 hours for three days. - Antibiotic therapy (when strictly indicated; avoid otherwise): a. First option, ciprofloxacin 500 mg – Take one tablet every 12 hours for three days; OR b. Second option, azithromycin 500 mg – Take one tablet a day for three days. |
| N94, N94.6 | Dysmenorrhea | - Dipyrone 1 g—take 2 tablets initially, followed by 1 tablet every 6 hours if pain persists, for up to 3 days. - Anti-inflammatories: (1) for moderate pain, mefenamic acid 500 mg—take 1 tablet every 8 hours if pain persists for up to 3 days; and (2) for severe pain, ketorolac trometamol 10 mg—take 2 tablets sublingually initially, followed by 1 tablet every 8 hours if pain persists, for up to 3 days. |
| K30 | Dyspepsia | - Antacid: (1) aluminum hydroxide 80 mg/mL, magnesium hydroxide 80 mg/mL, or simethicone 6 mg/mL—take 20 mL initially and then 10 mL 3 times daily between meals for up to 7 days. - Antiemetic: ondansetron 8 mg—take 1 tablet sublingually every 8 hours for nausea for up to 3 days. - Prokinetic: bromopride 10 mg—take 1 tablet every 8 hours for gastric fullness for 7 days. - Pump Proton Inhibitor: pantoprazole 40 mg—take 1 tablet daily on an empty stomach for up to 7 days. |
| M08, M16, M17, M18, M23, M24, M25, M25.5, M75, M77, M96, S53, S83, S93, Z96 | Articular pain | • Anti-inflammatories: (1) with a history of dyspepsia, naproxen 500 mg + esomeprazole 20 mg—take 1 tablet every 12 hours for pain for up to 7 days; (2) for moderate pain, ketoprofen 100 mg—take 1 tablet every 12 hours for pain for up to 3 days; (3) for severe pain, ketorolac trometamol 10 mg—take 2 tablets sublingually as a single dose and then, 1 tablet every 8 hours if pain persists for up to 3 days.  • Dipyrone 1 g—take 2 tablets initially, followed by 1 tablet every 6 hours if pain persists, for up to 7 days.  • Paracetamol 750 mg—take 1 tablet every 6 hours if pain persists for up to 7 days. |
| G71, M41, M62, M79.6, M95, M96, R25.2, R93, S86 | Muscle pain | • Anti-inflammatories: (1) with a history of dyspepsia: naproxen 500 mg + esomeprazole 20 mg—take 1 tablet every 12 hours for pain for up to 7 days; (2) for moderate pain; ketoprofen 100 mg—take 1 tablet every 12 hours for pain for up to 3 days; (3) for severe pain, ketorolac trometamol 10 mg—take 2 tablets sublingually as a single dose, followed by 1 tablet every 8 hours if pain persists, for up to 3 days.  • Dipyrone 1 g—take 2 tablets initially, followed by 1 tablet every 6 hours if pain persists, for up to 7 days.  • Cyclobenzaprine 5 mg—take 1 tablet before bedtime for 7 days.  • For those with allergies: paracetamol 750 mg—take 1 tablet every 6 hours if pain persists for up to 7 days. |
| B86 | Scabies | • Permethrin 5% cream—apply before bedtime from the neck down, thoroughly over the entire body, to the soles of the feet, including between the fingers and intimate parts (avoid mucous membranes). Rinse off during usual morning hygiene. Repeat treatment in 7 days if necessary.  • Ivermectin 6 mg—take 2 tablets as a single dose (or based on weight—approximately 0.2 mg/kg).  • Desloratadine 5 mg—take 1 tablet daily in the morning for 3 days.  • Hydroxyzine 25 mg—take 1 tablet daily before bedtime for 3 days.  • Hypoallergenic, fragrance-free moisturizing cream—apply to affected areas as needed, especially if itchiness occurs. |
| J02, J03, J06 | Pharyngotonsillitis | - Analgesic or antipyretic: dipyrone 1 g (preferably) or paracetamol 750 mg, or ibuprofen 400 mg. - Topical analgesia: hexomedine spray or benzidamine tablets. - Consider anti-inflammatories: nimesulide or ketoprofen. - Consider corticosteroid therapy: prednisolone 40 mg/day for 2-3 days. - Antibiotic therapy: (1) first option, amoxicillin for 10 days; (2) second option, cephalexin or cefadroxil for 10 days; (3) third option, azithromycin (consider using oral corticosteroids concurrently) or clindamycin; and (4) avoid sulfa or quinolone antibiotics. |
| M10 | Gout | • Anti-inflammatories: (1) with a history of dyspepsia, naproxen 500 mg + esomeprazole 20 mg—take 1 tablet every 12 hours for pain for up to 7 days; (2) for moderate pain: naproxen 500 mg—take 1 tablet every 12 hours if pain persists for up to 7 days; and (3) for severe pain, ketorolac trometamol 10 mg—take 2 tablets sublingually as a single dose and then, 1 tablet every 8 hours if pain persists for up to 3 days. If pain improves, switch to naproxen 500 mg every 12 hours for another 4 days to complete a 7-day treatment course.  • Dipyrone 1 g—take 2 tablets initially, followed by 1 tablet every 6 hours if pain persists, for up to 7 days.  • Colchicine 0.5 mg—take 2 tablets initially and then continue with 1 tablet every 8 hours for up to 3 days. Discontinue if diarrhea develops.  • Prednisolone 40 mg—take 1 tablet daily for 7 days. |
| I84, K60.2, K61, K60.3, L05, O22, O87 | Hemorrhoids | • Topical treatment (simple regimen): Ointment containing chinchocaine + polycresulene – Apply to affected areas 3 to 4 times a day for up to 5 days.  • Anti-inflammatories:   - With a history of dyspepsia: Naproxen 500 mg + esomeprazole 20 mg – Take one tablet every 12 hours for pain for up to 7 days. - For moderate pain: Ketoprofen 100 mg – Take 1 tablet every 12 hours if pain persists for up to 3 days. - For severe pain: Ketorolac trometamol 10 mg – Take 2 tablets sublingually as a single dose and then 1 tablet every 8 hours if pain persists for up to 3 days.   • Dipyrone 1 g—take 2 tablets initially, followed by 1 tablet every 6 hours if pain persists, for up to 7 days.  • For allergies: paracetamol 750 mg—take 1 tablet every 6 hours if pain persists for up to 7 days. |
| A60, B00, B02 | Herpes simplex | • Hypoallergenic lip moisturizer with sun protection—apply to affected areas 3 times daily for 3 days.  • Aciclovir cream 50 mg/g—apply a thin layer to affected areas every 4 hours until improvement.  • Oral aciclovir 200 mg—take 2 tablets every 8 hours for 7 days; OR  • Valacyclovir 500 mg—take 4 tablets as a single dose. |
| B34.2, B97 | COVID-19 | - Antihistamines + decongestants: desloratadine + pseudoephedrine, fexofenadine + pseudoephedrine for 7 days (consider levocetirizine + montelukast once daily for 7 days in the presence of a history of rhinitis or asthma or bronchitis, depending on availability). - For a predominantly dry cough at night, consider dexchlorpheniramine 2-6 mg or hydroxyzine 25 mg at night for 7 days. - If corticosteroids are indicated: prednisolone 40 mg/day for up to 7 days or nasal corticosteroids for 2 weeks. - Nasal irrigation with saline solution 5 times/day for 2 weeks. |
| M51, M54, S32, S33, S34 | Back pain | • Anti-inflammatories: (1) for moderate pain, ketoprofen 100 mg—take 1 tablet every 12 hours for pain for up to 3 days; and (2) for severe pain; ketorolac trometamol 10 mg—take 2 tablets sublingually as a single dose, followed by 1 tablet every 8 hours if pain persists, for up to 3 days; (30 with a history of dyspepsia, naproxen 500 mg + esomeprazole 20 mg—take 1 tablet every 12 hours or pain for up to 7 days.  • Dipyrone 1 g—take 2 tablets as a single dose, followed by 1 tablet every 6 hours if pain persists, for up to 7 days.  • Cyclobenzaprine 5 mg—take 1 tablet before bedtime for 7 days. |
| L55, T20-T31, T95 | Burns | • For indicated cases, topical antibiotic: bacitracin ointment or fusidic acid—apply a thin layer to affected areas twice a day for 5 days (avoid combining with corticosteroids).  • Hypoallergenic moisturizing cream, fragrance-free, rich in lipids—apply to affected areas twice a day for 5 days.  • Hydrocolloid dressing, 3 layers—apply to clean affected areas and change every 2-3 days (note: accumulation of a small amount of clear secretion on the dressing is normal).  • Dipyrone 1 g—take 2 tablets initially and then, 1 tablet every 6 hours for pain.  • Anti-inflammatories: (1) with a history of dyspepsia, naproxen 500 mg + esomeprazole 20 mg—take 1 tablet every 12 hours for pain for up to 7 days; (2) for moderate pain, naproxen 500 mg—take 1 tablet every 12 hours for pain for up to 7 days; and (3) for severe pain, ketorolac trometamol 10 mg—take 2 tablets sublingually as a single dose and then, 1 tablet every 8 hours if pain persists for up to 3 days. Upon improvement, switch to naproxen 500 mg every 12 hours for another 4 days to complete a 7-day treatment course. |
| J11 | Flu syndrome | - If symptoms of SARS^b^ occur, seek in-person care immediately. - Oseltamivir—orescribe with *ICD* code J11.8 for dispensing in referenced locations. - For at-risk individuals: initiate within 5 days of symptom onset (best within the first 24 to 48 hours). - Adults: oseltamivir (75 mg) every 12 hours for 5 days; adjust dosage if creatinine clearance is below 30 mL/min. - Children: determine dosage according to weight. - Treat symptoms according to the nasopharyngitis (common cold) protocol: analgesics/antipyretics, antihistamines, nasal irrigation, and nasal corticosteroids. - Avoid systemic anti-inflammatories and corticosteroids. - Home isolation (droplet precautions): adults for 5 days and children for 7 days (note: isolation of asymptomatic contacts is not necessary. - Testing for influenza A and B—do not request from contracted networks (may not be covered by insurance). - Collect a sample 48 hours after symptom onset. Additionally, request RT‒PCR for SARS-CoV-2 or a rapid antigen test for SARS-CoV-2 (COVID-19). |
| J01 | Sinusitis | - Antibiotics (not mandatory in all cases): (1) first option, amoxicillin with clavulanate for 7 days; (2) second option, cefuroxime 500 mg for 7 days; (3) third option (for severe penicillin allergy), levofloxacin 500 mg for 7 days; and (4) fourth option (avoid or justify if used); doxycycline—provide justification for extended use. - Macrolides or sulfonamides are not recommended. - Corticosteroids: (1) nasal: nometasone (Nasonex) 2 sprays in each nostril 2-3 times a day; (2) oral: prednisolone 40 mg/day for 4 days—avoid use extending beyond 7 days. - Analgesic or antipyretic: dipyrone 1 g—preferably—OR paracetamol 750 mg OR ibuprofen 400 mg. |
| G2.3, M43.6 | Torticollis | • Anti-inflammatories: (1) with a history of dyspepsia, naproxen 500 mg + esomeprazole 20 mg—take 1 tablet every 12 hours for pain for up to 7 days; and (2) for moderate pain, ketoprofen 100 mg—take 1 tablet every 12 hours for pain for up to 3 days; and (3) for severe pain, ketorolac trometamol 10 mg—take 2 tablets sublingually as a single dose, followed by 1 tablet every 8 hours if pain persists, for up to 3 days.  • Dipyrone 1 g—take 2 tablets as a single dose, followed by 1 tablet every 6 hours if pain persists, for up to 7 days.  • Cyclobenzaprine 5 mg—take 1 tablet before bed for 7 days.  • For those with allergies: paracetamol 750 mg—take 1 tablet every 6 hours for pain for up to 7 days. |
| R05 | Cough | - Antihistamines + decongestants: desloratadine + pseudoephedrine, fexofenadine + pseudoephedrine for 7 days (consider levocetirizine + montelukast once daily for 7 days in the presence of a history of rhinitis or asthma or bronchitis, depending on access). - If experiencing a predominantly dry cough at night, consider dexchlorpheniramine 2-6 mg or hydroxyzine 25 mg at night for 7 days. - If corticosteroids are indicated: prednisolone 40 mg/day for up to 7 days or nasal corticosteroids for 2 weeks. - As a therapeutic test: pantoprazole or esomeprazole 40 mg/day for up to 4 weeks (up to 8 weeks of total treatment) and recommend an in-person evaluation. - Nasal irrigation with saline solution 5 times/day for 2 weeks. |
| L50, L56 | Urticaria | - Antihistamine: (1) dexchlorpheniramine 2 mg—take 3 tablets initially and then, 1 tablet every 8 hours until improvement; OR (2) hydroxyzine 25 mg—take 2 tablets initially and then 1 tablet every 6 hours until improvement; OR (3) desloratadine 5 mg (for mild cases); OR (4) fexofenadine 180 mg—take 1 tablet daily for 5 days. - Corticosteroid: prednisolone 40 mg—take 1 tablet daily for 5 days. - H2 histamine receptor antagonist: famotidine 40 mg—take 1 tablet daily for 5 days. |
| A88, H81 | Vertigo | • Dimenhydrinate 50 mg—take 2 tablets initially and then, 1 tablet every 8 hours until improvement.  • Meclizine 25 mg—take 2 tablets initially and then, 1 tablet every 12 hours until improvement.  • Ondansetron 8 mg—take 1 tablet sublingually every 8 hours for nausea.  • Betahistine 16 mg—take 1 tablet every 8 hours for 5 days if symptoms persist. |
| N77, N77.1, B37.3 | Vulvovaginitis | - Topical treatment: (1) tioconazole 100 mg/5 g + tinidazole 150 mg/5 g—apply 1 dose vaginally before bedtime; avoid using tampons during treatment. - Oral fluconazole: fluconazole 150 mg—take 1 tablet as a single dose. |

^a^*ICD-10*: *International Statistical Classification of Disease, Tenth Revision*.

^b^SARS: severe acute respiratory syndrome.
